# Saddle Pulmonary Embolism Detected by Transthoracic Echocardiography in a Patient With Suspected Myocardial Infarction

**DOI:** 10.1016/j.case.2023.11.006

**Published:** 2023-12-15

**Authors:** Eugene Yuriditsky, James M. Horowitz, Bedros Taslakian, Muhamed Saric

**Affiliations:** a Division of Cardiology, Department of Medicine, NYU Langone Health, New York, New York; b Division of Vascular and Interventional Radiology, Department of Radiology, NYU Langone Health, New York, New York

**Keywords:** Pulmonary embolism, Transthoracic echocardiography, Thrombolysis

## Abstract

• PE is very rarely identified on TTE.
• Saddle PE does not represent a higher-risk subset of PE.
• Catheter-based therapies are becoming more commonplace in the management of acute PE.

## Graphical abstract

**Figure:**
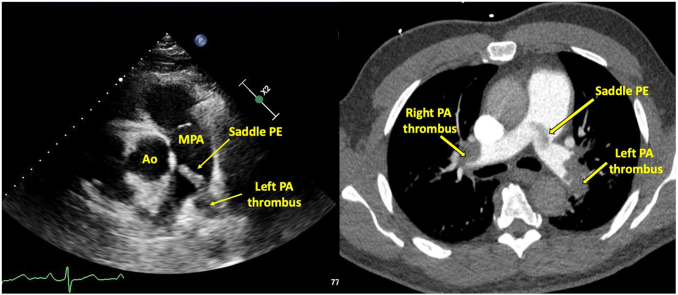

## Introduction

While transthoracic echocardiography (TTE) is not a first-line test in the diagnosis of acute pulmonary embolism (PE), indirect signs such as right ventricular (RV) dilatation and dysfunction may be a clue to the diagnosis and aid in risk stratification. Saddle PE, that is, one occurring at the bifurcation of the main pulmonary artery (PA), is very seldom detected via TTE; only a few case reports describe this finding.^1^^,^^2^ Acoustic windows often pose a limit to the visualization of the pulmonary trunk and bifurcation. Here we present a patient suspected to have acute coronary syndrome (ACS) diagnosed with saddle embolism by TTE.

## Case Presentation

A 59-year-old man with a history of hypertension and chronic kidney disease stage II presented to the emergency department with a week of exertional dyspnea. Several days prior, the patient was evaluated by a primary care physician for similar symptoms and prescribed azithromycin for a presumptive diagnosis of bronchitis. As symptoms continued to worsen, they sought medical attention in the emergency department. On initial evaluation, heart rate was 76 beats per minute, blood pressure was 111/73 mm Hg, and oxygen saturation of 97% on room air. The physical examination was benign: the patient was in no apparent distress, breathing comfortably, and was not diaphoretic; there was no lower extremity tenderness or swelling. The patient did not have a history of venous thromboembolism, did not suffer recent trauma, and did not have an active or prior malignancy.

**Figure 1:**
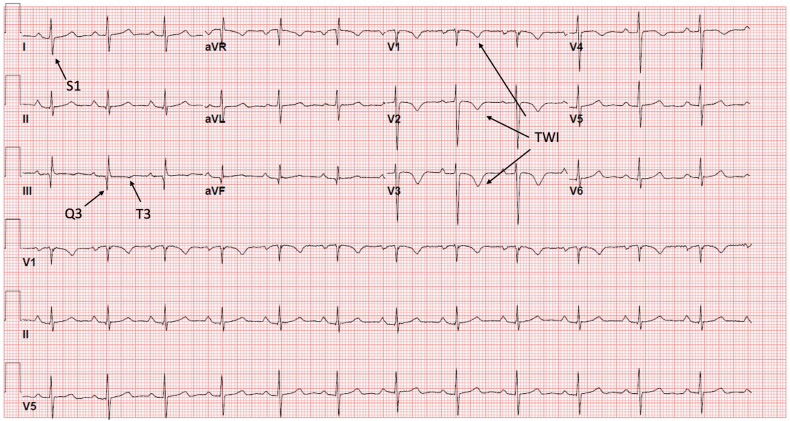
Electrocardiogram. Sinus rhythm with precordial T-wave inversions (TWI) and an S1Q3T3 pattern consistent with RV strain.

The electrocardiogram in triage demonstrated T-wave inversions in the precordial leads with S waves in lead I and Q waves along with T-wave inversions in lead III (Figure 1). Labs were significant for a troponin I of 0.14 ng/mL (normal, <0.08), brain natriuretic peptide of 534 pg/mL (normal, <100), and serum creatinine of 1.55 mg/dL (normal range, 0.7-1.3). Chest x-ray did not demonstrate pneumothorax, pleural effusion, or signs of parenchymal disease.

As the electrocardiogram demonstrated precordial T-wave inversions in this patient without active chest discomfort, a diagnosis of Wellen’s syndrome was made by emergency providers. The patient was therefore treated for a non-ST segment elevation myocardial infarction with aspirin, clopidogrel, and heparin. An echocardiogram was ordered by emergency physicians to define left ventricular (LV) systolic function, and cardiology was consulted to evaluate the patient for coronary angiography.

**Figure 2:**
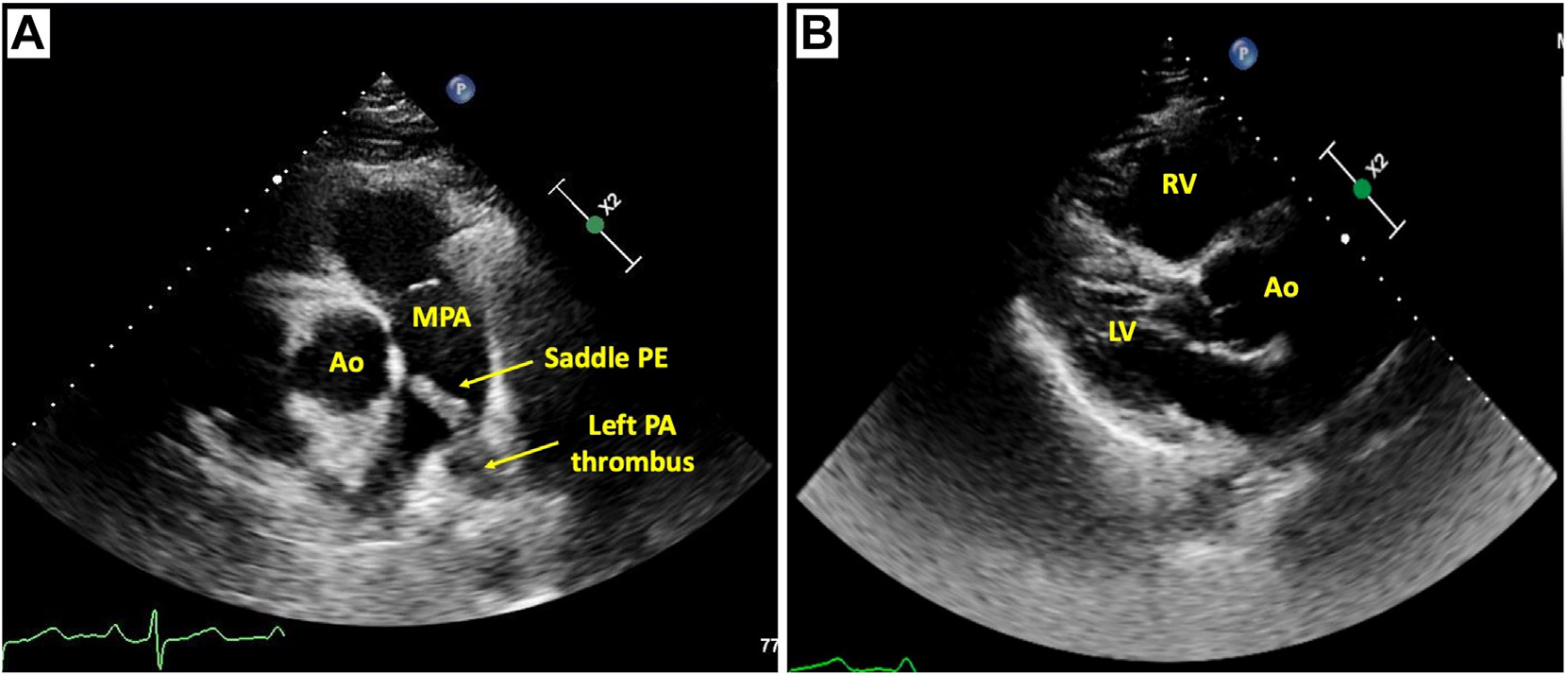
Transthoracic echocardiogram: saddle pulmonary embolism. Parasternal short-axis view of the bifurcation of the PA in diastole. Left PA PE extending to the main PA is demonstrated **(A)**. Parasternal long-axis view in diastole demonstrating RV dilatation (proximal RV outflow tract diameter, 4.0 cm; normal range, 2.1-3.5 cm) **(B)**. *Ao*, Aorta; *MPA*, main PA.

**Figure 3:**
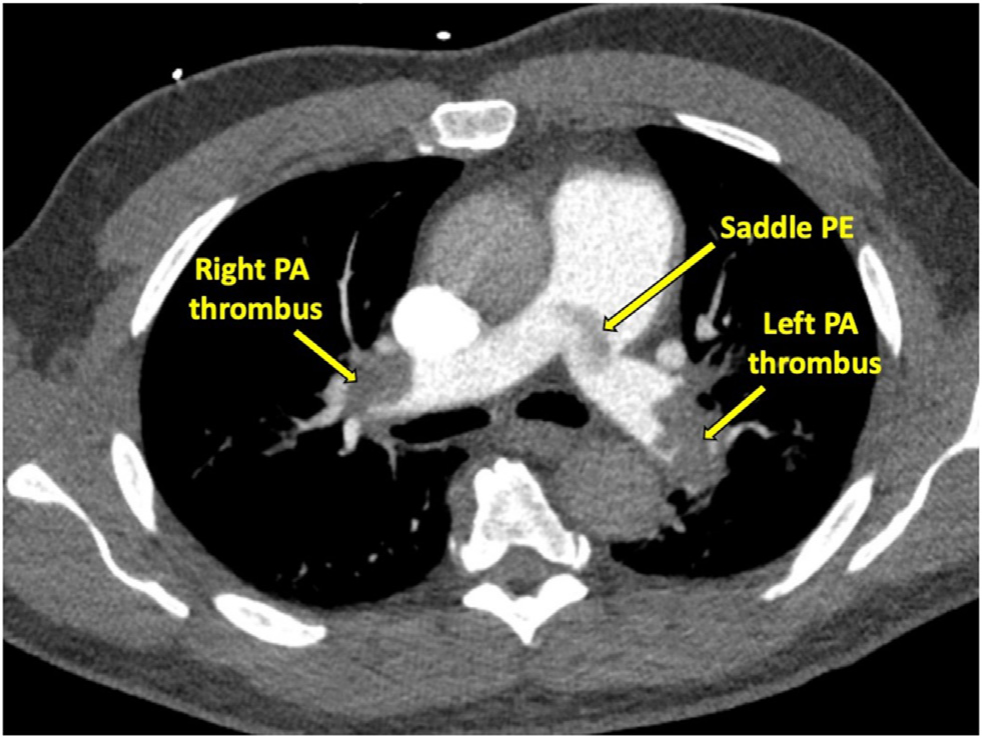
Computed tomography: saddle pulmonary embolism. Bilateral PEs and a saddle PE are demonstrated corresponding to the echocardiographic view. The main PA is dilated to 3.7 cm, suggestive of chronicity.

Prior to cardiology evaluation, the patient was admitted to a telemetry unit and a TTE was performed. On echocardiography, the right ventricle (RV) was dilated and hypokinetic with preserved LV systolic function. The RV base was measured at 5.1 cm (normal range, 2.5-4.1 cm), RV mid diameter was 4.0 cm (normal range, 1.9-3.5 cm), and proximal RV outflow tract measured 4.0 cm (normal range, 2.1-3.5 cm).^3^ The tricuspid annular plane systolic excursion (TAPSE) was 1.1 cm (abnormality threshold, <1.7 cm), and the tricuspid regurgitation maximum velocity was 4.0 m/sec (estimated RV systolic pressure = 72 mm Hg).^3^ A linear mobile echodensity consistent with thrombus was visualized at the bifurcation of the main PA (Figure 2, Video 1). Coronary angiography was therefore deferred, and a computed tomography (CT) pulmonary arteriogram was performed that demonstrated a dilated main PA (3.7 cm) and saddle PE in addition to extensive bilateral emboli involving all lobes and extending into the segmental and subsegmental vessels (Figure 3).

**Figure 4:**
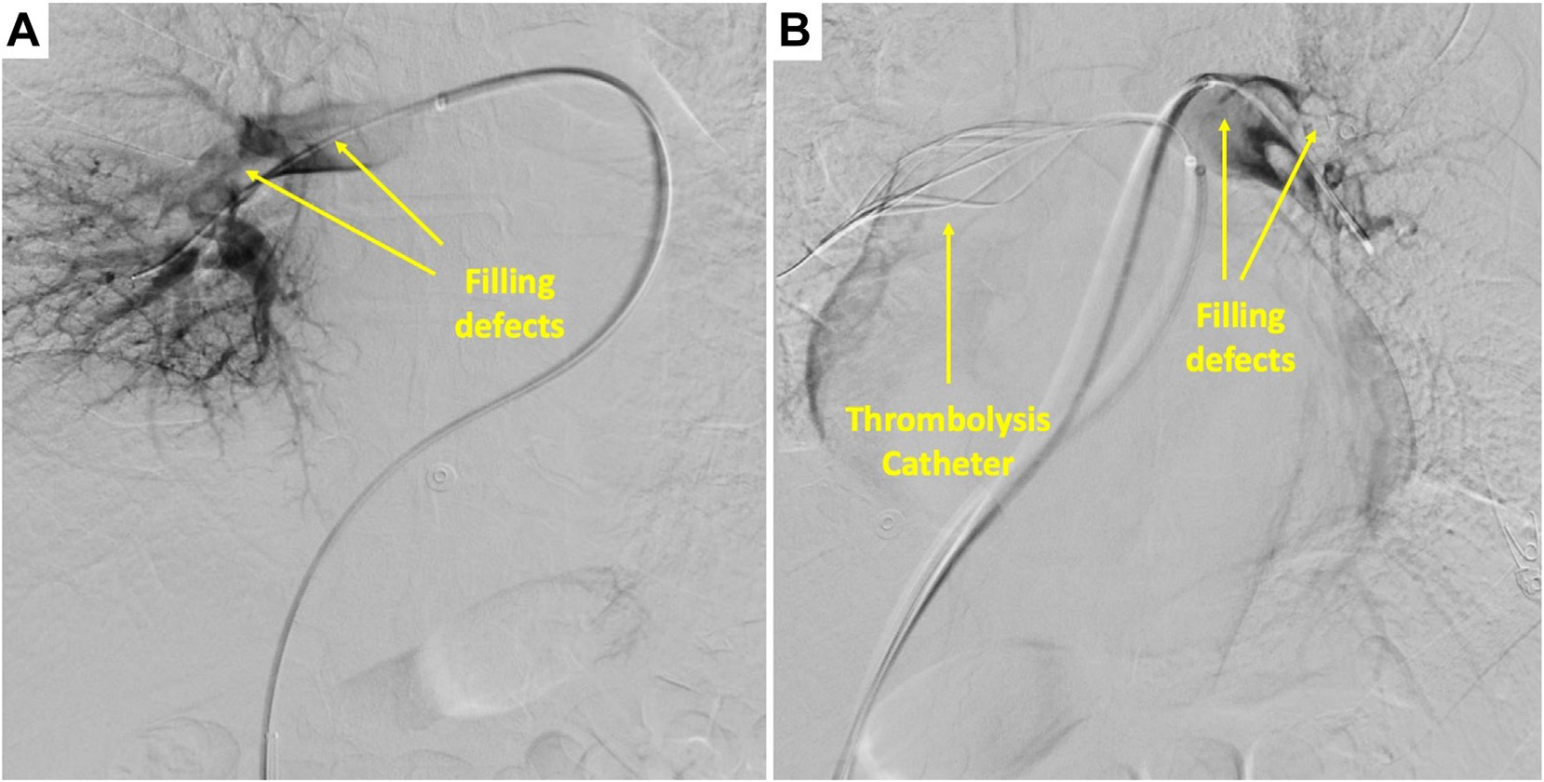
Right PA angiogram with filling defects consistent with pulmonary emboli **(A)**. Left pulmonary angiogram with filling defects with the thrombolysis catheter in the right PA **(B)**.

Due to persistent symptoms and significant clot burden, the patient underwent catheter-directed thrombolysis. Following pulmonary angiography, thrombolysis catheters were expanded in the bilateral PAs with tissue plasminogen activator infused at a rate of 1 mg per hour over 5 hours (Figure 4, Video 2). Postprocedure, the patient had an uneventful hospital course, described much improved dyspnea with ambulation, and was discharged on apixaban in 48 hours.

## Discussion

We present a patient with an unprovoked, intermediate- to high-risk PE (imaging evidence of RV dysfunction and abnormal cardiac laboratory biomarkers) initially masquerading as a non-ST segment elevation myocardial infarction. A saddle PE was identified by TTE prior to confirmation by CT angiography. The electrocardiographic RV injury pattern with precordial T-wave inversions is a known mimic of ACS.^4^ However, patients with ACS are more likely to have T-wave inversions extending through leads V5-6 (absent in our patient), while patients with PE were more likely to have T-wave inversions in the inferior leads (present in lead III in our patient).^4^ The absence of tachycardia in our patient is interesting and may have dissuaded the initial pursuit of the diagnosis of acute PE. However, tachycardia is found in approximately 38% of cases of acute PE and the lower heart rate may have been suggestive of some degree of chronicity.^5^

Saddle PE is a rare finding on TTE, with few case reports published.^1^^,^^2^^,^^6^^,^^7^ The pulmonary trunk may be visualized in the parasternal short-axis view with the probe fanned cephalad.^8^ In this view, the pulmonic valve and PA and its branches are assessed in addition to proximal and distal RV outflow tract segments.^8^ The detection of thrombus in this view may be challenging due to poor acoustic windows. In our patient, increased clot echogenicity, at times associated with chronicity, may have aided visualization. Among patients too unstable for CT with suboptimal TTE findings to fully assess RV dilatation/dysfunction, visualization of PA thrombus may assist in the diagnosis of PE.

The subcostal short-axis of the basal RV provides similar information when the parasternal short-axis view is limited.^8^ A modified right parasternal view may allow for the visualization of the distal right PA branch. With the patient in a steep left lateral decubitus position, the right PA is examined with the transducer at 30° to 45° to the chest wall at the second intercostal space immediately to the right of the sternum.^9^ The left PA branch is challenging to visualize both by TTE and transesophageal echocardiography as it is shielded by the left main stem bronchus.^10^ However, as the left PA dilates, it may be more readily seen as the window becomes more available around the acoustic limitations.

Echocardiographic signs are highly specific while lacking sensitivity for the diagnosis of acute PE among patients without underlying cardiopulmonary disease.^11^ In a large meta-analysis, “right heart strain” (defined as RV dysfunction, RV strain, an abnormal right heart, or cor pulmonale) was 83% specific but only 53% sensitive for the diagnosis of acute PE.^11^ McConnell’s sign (RV free wall akinesis with apical sparing) is commonly associated with acute PE and is 97% specific while only 22% sensitive for the diagnosis.^11^^,^^12^ Following its initial description, subsequent studies noted this finding to be less specific than initially thought.^12^ The “60/60 sign,” defined as a tricuspid regurgitation jet gradient of <60 mm Hg and a pulmonary acceleration time of >60 msec, is more objective and reproducible than McConnell’s sign and carries a pooled specificity of 84%.^11^ The sensitivity of TTE among more critical patients with higher pretest probability may be improved.

Beyond its use in the diagnosis of acute PE, echocardiographic findings carry prognostic value. Tricuspid annular plane systolic excursion, a well-known and relatively simple measure, along with several additional markers of RV systolic function, have been shown to inconsistently predict PE-related mortality.^12^ More recently, the LV outflow tract velocity-time integral, a stroke volume surrogate, was demonstrated to predict short-term outcomes even among initially normotensive patients.^13^ Thrombus in transit, due to its propensity to cause immediate hemodynamic instability, carries a high mortality rate, particularly without therapy.^12^ Accordingly, echocardiographic findings may inform on prognosis and assist in selecting patients for interventional therapies.

Saddle PE accounts for <5% of all PE cases. Before widespread use of CT angiography, this finding was often postmortem and therefore synonymous with a severe, hemodynamically significant presentation. However, several studies have dispelled this assertion, demonstrating similar clinical outcomes among those with and without saddle PE.^14^^,^^15^ Saddle emboli may be less flow limiting, with distal clot burden having more impact on RV function.^14^

With the evolution of catheter-based therapies, the management of PE has seen a revolution over the past decade. Often coupled with mechanisms allowing for thrombus fragmentation, catheter-directed thrombolysis allows for the delivery of tissue plasminogen activator directly into the clot at a fraction of the usual systemic dose.^16^ Observational studies demonstrate improved surrogate end points among patients with intermediate- and high-risk PE.^17^ As our patient with intermediate- to high-risk PE had persistent symptoms despite systemic anticoagulation, catheter-directed thrombolysis was selected as the next management step, leading to a significant subjective improvement. With several randomized control trials underway, we hope to see appropriate use criteria better defined for interventional therapies in acute PE.

## Conclusion

The echocardiographic assessment of RV function factors into risk stratification among patients with acute PE. However, saddle PE is rarely detected via TTE and only a few case reports describe this finding. While historically thought to be a harbinger of a more severe presentation, recent studies prove saddle emboli are associated with similar short-term outcomes as nonsaddle PE.

## Consent Statement

The authors declare that since this was a non-interventional, retrospective, observational study utilizing de-identified data, informed consent was not required from the patient under an IRB exemption status.

## Ethics Statement

The authors declare that the work described has been carried out in accordance with The Code of Ethics of the World Medical Association (Declaration of Helsinki) for experiments involving humans.

## Funding Sources

This research did not receive any specific grant from funding agencies in the public, commercial, or not-for-profit sectors.

## Conflict of Interest

The authors report no conflict of interest.

## Supplementary Data

Video 1 Serial display of three two-dimensional TTE views. Modified parasternal short-axis view demonstrates the mobile thrombus within the main PA and extending into the left PA and right PA (saddle embolus). The parasternal long-axis view demonstrates the small LV with normal LV systolic function, the dilated RV outflow tract, and thick moderator band. The apical 4-chamber view demonstrates the small LV with normal LV systolic function and the dilated RV (base = 5.1 cm) with reduced RV systolic function (TAPSE = 1.1 cm).

Video 2 Right and left pulmonary angiograms demonstrate filling defects within the PAs (PE) and the thrombolysis catheter positioned within the right PA.
